# Oral sodium bicarbonate in people on haemodialysis: a randomised controlled trial

**DOI:** 10.1186/s12882-021-02549-x

**Published:** 2021-10-21

**Authors:** Stella I. Kourtellidou, Damien R. Ashby, Lina R. Johansson

**Affiliations:** 1 Department of Nutrition and Dietetics, Hammersmith Hospital, Imperial College Healthcare NHS Trust, Du Cane Road W12 0HS, London, UK; 2 Renal and Transplant Centre, Hammersmith Hospital, Imperial College Healthcare NHS Trust, Du Cane Road W12 0HS, London, UK

**Keywords:** Sodium bicarbonate, Haemodialysis, Potassium, Muscle

## Abstract

**Background:**

Adverse events and mortality tend to cluster around dialysis sessions, potentially due to the impact of the saw-toothed profile of uraemic toxins such as potassium, peaking pre-dialysis and rapidly dropping during dialysis. Acidosis could be contributing to this harm by exacerbating a rise in potassium. The objectives of this study were to investigate the effects of oral bicarbonate treatment on reducing inter-dialytic potassium gain as well as other clinical consequences of preserving muscle mass and function and reducing intradialytic arrhythmia risk in people on haemodialysis.

**Methods:**

Open-label randomised controlled trial in a single-centre (London, UK). Forty-three clinically stable adults on haemodialysis were recruited, with a 6 month average pre-dialysis serum bicarbonate level < 22 mmol/l and potassium > 4 mmol/l. Thirty-three participants completed the study. Oral sodium bicarbonate tablets titrated up to a maximum of 3 g bd (6 g total) in intervention group for 12 weeks versus no treatment in the control group. Outcomes compared intervention versus non-intervention phases in the treated group and equivalent time points in the control group: pre- and post-dialysis serum potassium; nutritional assessments: muscle mass and handgrip strength and electrocardiograms (ECGs) pre and post dialysis.

**Results:**

Participants took an average of 3.7 ± 0.5 g sodium bicarbonate a day. In the intervention group, inter-dialytic potassium gain was reduced from 1.90 ± 0.60 to 1.69 ± 0.49 mmol/l (*p* = 0.032) and pre-dialysis potassium was reduced from 4.96 ± 0.62 to 4.79 ± 0.49 mmol/l without dietary change. Pre-dialysis bicarbonate increased from 18.15 ± 1.35 to 20.27 ± 1.88 mmol/l, however with an increase in blood pressure. Nutritionally, lean tissue mass was reduced in the controls suggesting less catabolism in the intervention group. There was no change in ECGs. Limitations are small sample size and unblinded study design lacking a placebo, with several participants failing to achieve the target of 22 mmol/l serum bicarbonate levels due mainly to tablet burden.

**Conclusion:**

Oral sodium bicarbonate reduced bicarbonate loss and potassium gain in the inter-dialytic period, and may also preserve lean tissue mass.

**Trial registration:**

The study was registered prospectively on 06/08/2015 with EU Clinical Trials Register EudraCT number 2015-001439-20.

## Background

The intermittent nature of haemodialysis (HD) allows uraemic   toxins to build up during the inter-dialytic period, with rapid removal during the dialysis session, resulting in a saw-toothed profile for some uraemic toxins. Unsurprisingly, adverse events, and even mortality (including sudden death) are found to cluster around the dialysis session [1, 2]. Methods to even out the saw-toothed profile are considered beneficial to delivering quality dialysis such as is seen with increased frequency HD.

Uraemic toxins closely fitting the saw-toothed profile include electrolytes, such as potassium, which is plausibly and statistically associated with peri-dialytic morbidity and mortality [3–6]. Acidosis follows a similar inverse pattern, with the bicarbonate levels gradually falling during the inter-dialytic period, before rapid supplementation during dialysis. Observational studies of clinical outcome support the view that acidosis is harmful, showing that, after adjustment for comorbidity, pre-dialysis bicarbonate levels below 22 mmol/L are associated with excess mortality [7, 8]. Acidosis is a likely contributor to peri-dialytic harms, for example by exacerbating the rise in plasma potassium levels [9], as well as having longer term adverse effects, such as increased muscle catabolism by impairing insulin and insulin-like growth factor-1 signalling which leads to muscle protein breakdown [10, 11]. The data for the association between acidosis and impaired muscle strength is conflicting with positive relationships found in a large cohort study of older people [12] yet negative findings of handgrip strength in a small pilot study of bicarbonate supplementation in people with moderate to advanced kidney disease [13]. No studies have investigated the relationship between acidosis and handgrip strength in people on HD.

Studies of acidosis treatment in people on haemodialysis typically focus on dialytic supplementation [14–16] which features abrupt correction of bicarbonate levels and incremental development of acidosis in the inter-dialytic period, however if given orally throughout the inter-dialytic period, uraemic acidosis could be continuously corrected, potentially reducing peri-dialytic and longer term harms. Studies to date in people on haemodialysis have been limited by small sample size and lacking in a control group. We hypothesised that oral bicarbonate treatment would reduce inter-dialytic potassium gain, preserve muscle mass and function and reduce intradialytic arrhythmia risk in people on haemodialysis.

## Methods

The aim of this study is to understand the clinical impact of oral sodium bicarbonate supplementation on pertinent dialysis-related trends: inter-dialytic potassium gain, muscle mass and function loss and the risk of arrhythmias in people on haemodialysis.

### Study design, population and setting

This was a single centre, open-label randomised controlled trial, involving a mixed ethnicity haemodialysis population in London, UK. Stable adult patients (aged 18-80 years), on haemodialysis for at least 3 months, were eligible for recruitment if they had 6-month average predialysis serum bicarbonate level below 22 mmol/l and potassium over 4 mmol/l. Those with recurrent hospital admissions, dementia or who were bedbound, were excluded.

### Intervention

**Fig. 1:**
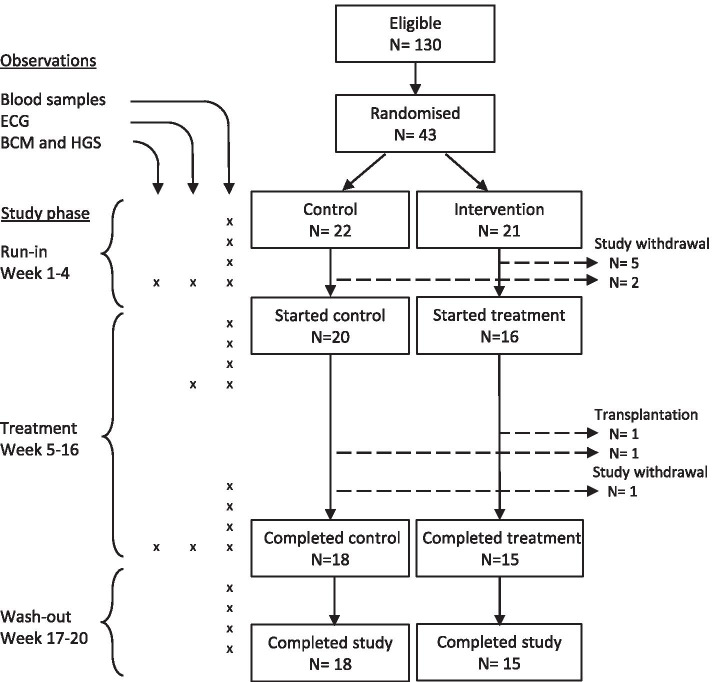
Patient flow through the study. The number of participants is provided at each stage, and reasons for non-completion. Blood samples, ECG and symptom data were primarily analysed in the intervention group, comparing treatment with run-in / wash-out phases in those starting treatment. Changes in BCM were compared between intervention and control groups in those completing the study. (ECG = electrocardiogram; BCM = Body Composition Monitor, Fresenius; HGS = Hand grip strength)

Participants were randomly allocated in 1:1 ratio, to intervention or control group by sealed envelopes by an individual outside the research team, stratified for gender and diabetes. Participants were enrolled by SK. The intervention group received a 4 week run-in phase (no treatment), followed by treatment for 12 weeks and finished with a 4 week wash-out phase, during which no treatment was given (Fig. 1). The control group received no treatment throughout all study phases. Treatment with sodium bicarbonate was started at 1 g bd (4 tablets per day), with the dose titrated during the first 4 weeks of treatment (increasing by 0.5 g bd as tolerated, to a maximum of 3 g bd) to achieve predialysis bicarbonate over 22 mmol/l. All patients were dialysing thrice weekly, using a standardised dialysate bicarbonate concentration of 35 mmol/l (final dialysate bicarbonate 32 mmol/l with acetate 3 mmol/l), and individualised dialysate potassium (either 1 mmol/l or 2 mmol/l). The clinical team were not restricted from altering the dialysate potassium as necessary for the duration of the study.

### Outcome measures

The primary outcome was inter-dialytic potassium gain. Secondary outcomes included changes in nutritional parameters (nutritional status and intake, muscle mass and handgrip strength) and electrocardiogram (ECG) parameters. Observations were conducted at time-points outlined in Fig. 1. Blood samples were taken after the long inter-dialytic interval for pre- and post-dialysis electrolytes. Standard clinical data including pre-dialysis blood pressure, inter-dialytic weight gain and dialysate potassium concentration were also recorded. ECGs were recorded pre- and post-dialysis at specific time points using MAC 1200ST ECG machines (GE Medical Systems) with standard software. Automated analysis was available for most parameters including QTc (calculated with the Bazett formula) but not QT dispersion, which was measured manually.

The nutritional assessments that were undertaken were: (1) nutritional status by the validated 7-point Subjective Global Assessment (SGA) [17] undertaken during dialysis sessions; (2) body composition (lean tissue mass and fat mass) and fluid overload by Body Composition Monitor (Fresenius) immediately prior to dialysis sessions; (3) muscle strength using Jamar hand dynamometer on the dominant / non-fistula arm (in the standing position) immediately prior to dialysis sessions; and (4) nutritional intake using 3-day food diaries covering 3 consecutive days including one weekend day, analysed using Diet Plan 6.0.

Adherence to the prescribed dose of sodium bicarbonate was assessed during titration and at the end of the study using medication diaries and investigator checks of remaining capsules.

### Statistical analysis

Differences between and within groups were compared using the t-test and chi-squared tests as appropriate, using SPSS version 23.0 (IBM, New York). The sample size calculation was based on potassium changes within the intervention group. We selected 0.3 mmol/l as a clinically meaningful change in pre-dialysis potassium and therefore the minimum change which the study should detect. Using an estimate of 0.6 mmol/l for the within-patient standard deviation of pre-dialysis potassium, the standard deviation of an average over 4 weeks would be 0.3 mmol/l. To achieve 90% power to detect a 0.3 mmol difference in pre-dialysis potassium with a significance level of 0.05 would require 21 per group, and we therefore aimed to recruit 25 participants in each group.

Electrolyte, ECG and blood pressure data were analysed both between groups, and within the intervention group, comparing final dose treatment (all timepoints after titration) with no treatment (run-in and wash-out periods). Typical dialysis-induced ECG changes were taken from the control group, across all timepoints.

Changes in nutritional parameters (body composition, muscle strength, nutritional status and intake) were compared both between groups (change during study) and within groups (study end vs baseline) in participants who completed the study.

Participants were analysed as randomised, excluding those who withdrew during the run-in phase, before any treatment initiation.

## Results

### Study recruitment and dose titration

From three satellite units providing haemodialysis for 518 patients, 130 (25.1%) were eligible and approached for enrolment (11/2015-12/2015) of which 43 participants (aged 27-79, 76% male) were recruited. Follow up was completed by May 2016. After randomisation 7 participants withdrew from the study (2 from the control arm and 5 from the intervention arm) during the run-in phase, leaving 16 (aged 27–74, 75% male) starting the intervention in week 5, and 20 starting the same timepoint in the control group (Fig. 1). The control and intervention arms were not significantly different to each other in their baseline demographic and clinical characteristics (Table 1). Two participants, one from each group, received a transplant during the treatment phase of the study and a further participant withdrew from the control group during the equivalent of the treatment phase. After dose titration participants in the intervention arm were taking an average sodium bicarbonate dose of 3.7 ± 0.5 g/day. The target bicarbonate level of 22 mmol/L was not consistently achieved (> 50% of readings) in 9/16 participants mainly due to tablet burden.

**Table 1:** Baseline characteristics of participants

	Control (*N* = 20)	Intervention (*N* = 16)	*p*-value
Age	58 (52-67)	57 (49-63)	0.18
Gender (male)	15 (75%)	12 (75%)	0.87
Ethnicity			0.26
White	8 (40%)	4 (25%)	
Black African / Caribbean	4 (20%)	5 (31%)	
Asian / Other	8 (40%)	7 (44%)	
Cause of renal disease			0.79
Diabetes	7 (35%)	5 (31%)	
Nephritis	6 (30%)	3 (19%)	
Hereditary	1 (5%)	2 (12%)	
Other	6 (30%)	6 (37%)	
Diabetes	9 (45%)	6 (38%)	0.83
Haemodialysis vintage (months)	38 (23-83)	23 (10-51)	0.14
Kt/V	1.6 (0.3)	1.44 (0.6)	0.65

Results given as median (IQR) or number (%)

### Electrolytes data

Compared to the control group, during the treatment weeks 13-16, inter-dialytic bicarbonate loss was lower in the treatment group (−4.05 ± 1.48 vs −6.52 ± 1.64, *p* < 0.001) and pre-dialysis bicarbonate was higher (20.10 ± 19.1 vs 16.77 ± 1.83, p < 0.001). But this was not accompanied by clear between group differences in inter-dialytic potassium gain (1.72 ± 0.55 vs 2.03 ± 0.60, *p* = 0.13) or pre-dialysis potassium (4.78 ± 0.55 vs 5.15 ± 0.62, *p* = 0.08, Table 2). Within the intervention group, compared to no treatment, inter-dialytic bicarbonate loss was reduced from 6.45 ± 1.91 to 4.12 ± 1.53 mmol/l on final dose treatment, leading to increased pre-dialysis bicarbonate levels (20.27 ± 1.88 vs 18.15 ± 1.35 mmol/l, *p* < 0.001 for both). Alongside this inter-dialytic potassium gain was reduced from 1.90 ± 0.60 to 1.69 ± 0.49 mmol/l (*p* = 0.032) leading to a possible reduction in pre-dialysis potassium (4.96 ± 0.62 to 4.79 ± 0.49 mmol/l, *p* = 0.07, Table 2). Food diaries suggested similar dietary potassium intake at the start and end of the treatment phase (27.6 ± 9.8 vs 29.2 ± 10.9 mg/kg/day, *p* = 0.34) suggesting that potassium gain was reduced primarily by intracellular redistribution (Table 3).

**Table 2:** Treatment dose and electrolytes in intervention and control groups

		No treatment		Week 5-8			Week 13-16			Final dose		
		Mean	SD	Mean	SD	p value^a^	Mean	SD	p value^a^			p value^b^
**Intervention (N = 16)**												
Bicarbonate dose (g/day)		0.0	0.0	2.2	0.3		3.7	0.5		3.7	0.5	
Potassium	Pre HD	4.96	0.62	4.83	0.40	0.038*	4.78	0.55	*0.08*	4.79	0.49	*0.07*
	Post HD	3.06	0.37	3.22	0.24	*0.63*	3.07	0.36	*0.61*	3.21	0.32	*0.70*
	ID change	1.90	0.60	1.61	0.29	*0.08*	1.72	0.55	*0.13*	1.69	0.49	0.032*
Bicarbonate	Pre HD	18.15	1.35	20.03	1.93	0.002*	20.10	1.91	< 0.001*	20.27	1.88	< 0.001*
	Post HD	24.59	1.67	24.77	1.38	*0.05*	24.15	1.00	*0.08*	24.39	1.23	*0.66*
	ID change	−6.45	1.91	−4.73	1.97	*0.12*	−4.05	1.48	< 0.001*	− 4.12	1.53	< 0.001*
Dialysate K = 1 (%)^c^		39.1		34.4		*0.71*	40.0		*0.74*	40.3		*0.88*
**Control (N = 20)**												
Potassium	Pre HD	5.22	0.60	5.24	0.69		5.15	0.62				
	Post HD	3.14	0.35	3.27	0.42		3.13	0.31				
	ID change	2.08	0.63	1.97	0.76		2.03	0.60				
Bicarbonate	Pre HD	18.02	1.55	18.16	1.47		16.77	1.83				
	Post HD	24.07	1.21	23.89	1.23		23.29	1.62				
	ID change	−6.05	1.35	−5.73	1.77		−6.52	1.64				
Dialysate K = 1 (%)^c^		38.8		43.4			36.1					

Except where stated, measurements are in mmol/l

HD haemodialysis

ID inter-dialytic interval

^a^Between groups (intervention vs control)

^b^Within intervention group (final dose vs no treatment)

^c^Proportion with dialysate potassium = 1 mmol/l (2 mmol/l standard)

*Significant at *p* < 0.05

**Table 3:** Nutritional outcomes in participants who completed the study

		Baseline		Study end			Change during study		
		Mean	SD	Mean	SD	p value^a^	Mean	SD	p value^b^
**Intervention (*N*** **= 15)**									
Body composition									
Dry weight (kg)		84.8	18.7	85.6	18.6	*0.05*	+ 0.9	1.6	*0.08*
Lean tissue mass (%)		45.0	12.2	45.5	12.5	*0.58*	+ 0.5	3.3	*0.07*
Fat mass (%)		38.8	9.4	38.3	9.8	*0.40*	−0.5	2.2	*0.29*
Over-hydration (L)		1.2	2.4	1.4	2.0	*0.60*	+ 0.2	1.4	*0.20*
Muscle strength									
Handgrip (kg)		26.6	13.1	26.2	13.4	*0.64*	−0.4	3.4	*0.32*
Nutritional intake									
Energy (kcal/kg/d)		21.5	6.8	22.6	7.8	*0.36*	+ 1.1	4.4	*0.79*
Protein (g/kg/d)		0.94	0.39	0.96	0.37	*0.58*	+ 0.02	0.14	*0.76*
nPCR (g/kg/d)		1.12	0.32	1.14	0.23	*0.72*	+ 0.02	0.21	*0.65*
Potassium (mg/kg/d)		27.6	9.8	29.2	10.9	*0.34*	+ 1.7	4.5	*0.06*
**Control (*N*** **= 18)**									
Body composition									
Dry weight (kg)		77.9	15.6	77.7	15.9	*0.64*	−0.2	1.7	
Lean tissue mass (%)		51.8	13.7	50.2	12.1	0.041*	−1.6	2.9	
Fat mass (%)		33.4	9.9	33.8	9.3	*0.52*	+ 0.4	2.5	
Over-hydration (L)		1.7	1.7	2.4	2.0	0.001*	+ 0.7	0.8	
Muscle strength									
Handgrip (kg)		31.2	10.9	29.7	10.1	0.034*	−1.5	3.0	
Nutritional intake									
Energy (kcal/kg/d)		22.7	9.4	24.3	9.3	*0.35*	+ 1.6	6.0	
Protein (g/kg/d)		0.92	0.35	0.91	0.32	*0.95*	−0.01	0.28	
nPCR (g/kg/d)		1.06	0.20	1.12	0.23	*0.36*	+ 0.06	0.21	
Potassium (mg/kg/d)		30.3	11.9	28.2	10.6	*0.23*	−2.1	6.7	

^a^Within groups (study end vs baseline)

^b^Between groups (change during study in intervention vs control group)

nPCR: normalised protein catabolic rate.

*Significant at *p* < 0.05

Potassium gain was therefore reduced by 0.21 mmol/l during bicarbonate treatment, without dietary change, despite only 44% achieving target bicarbonate levels. The size of this effect can be appreciated by comparison with the effect of altering potassium concentration in the dialysate: dialysate concentrates during the study were adjusted by clinical staff according to standard protocols, using a dialysate potassium of either 1 mmol/l or 2 mmol/l. The selection of the 1 mmol/l rather than 2 mmol/l dialysate potassium was associated with a greater intra-dialytic potassium reduction of 2.17 vs 1.73 mmol/l (*p* < 0.001). Therefore the 1 mmol/l dialysate potassium increased potassium removal by 0.43 mmol/l (findings not presented).

**Fig. 2:**
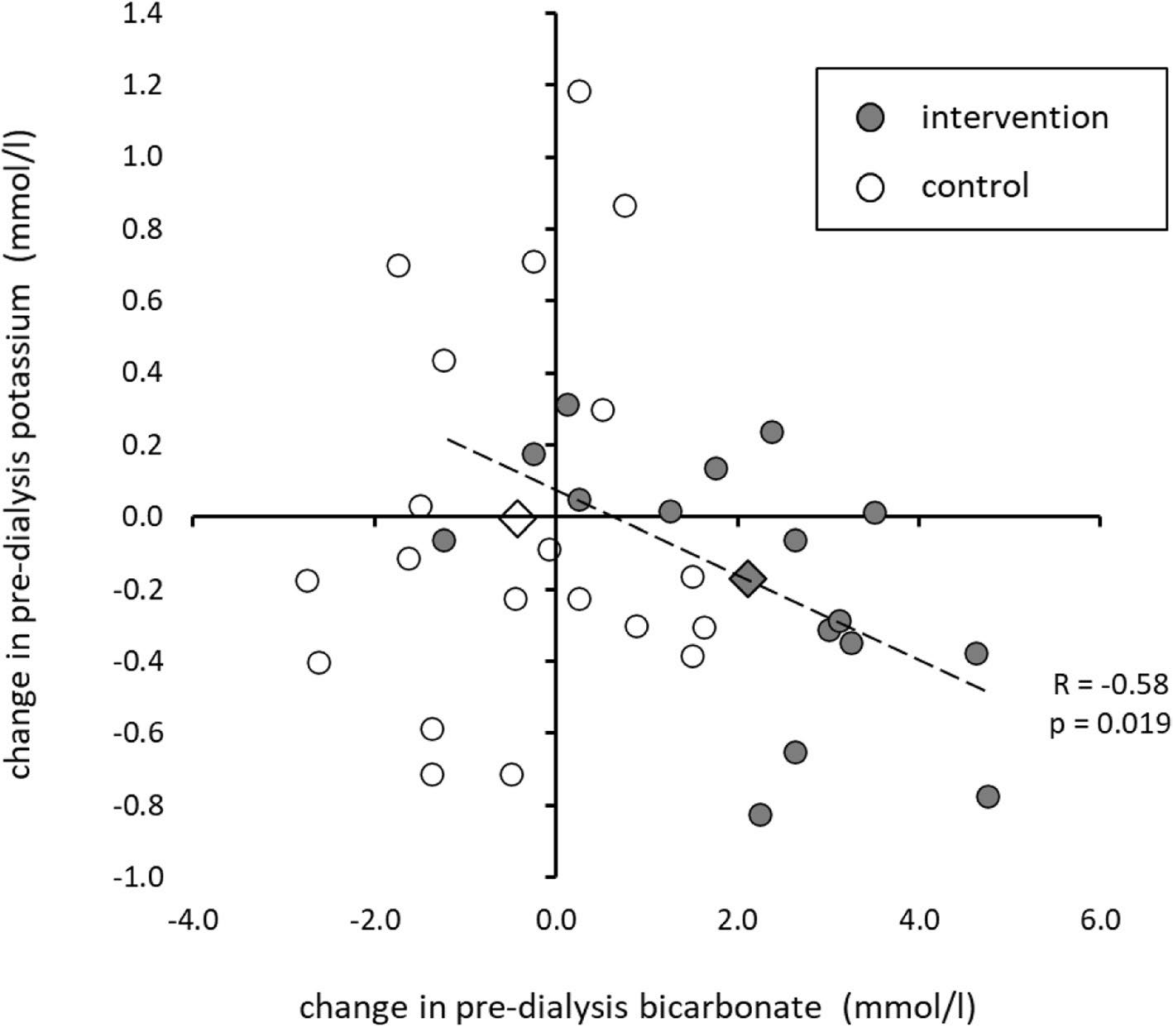
Relationship between the effect of oral sodium bicarbonate on pre-dialysis bicarbonate and the effect on pre-dialysis potassium. Individual summary data are shown as well as the group averages (diamonds). There was no change in bicarbonate or potassium in control group (white). In the intervention group (grey) the reduction in pre-dialysis potassium during treatment, was dependent on an increase in pre-dialysis bicarbonate

The relationship between pre-dialysis bicarbonate and potassium levels is shown in Fig. 2. Changes in potassium strongly correlated with changes in bicarbonate (R = − 0.58, *p* = 0.019) suggesting a dose-response effect, with pre-dialysis potassium reduced by approximately 0.1 mmol/l for every 1 mmol/l increase in pre-dialysis bicarbonate. Of particular importance, the frequency of clinically relevant hyperkalaemia (≥6.0 mmol/l) was reduced from 11.3 to 3.9% of pre-dialysis measurements (*p* = 0.037) whilst the hyperkalaemia frequency remained consistent in the control group at approximately 13.8% of measurements.

### Nutritional parameters

The whole group were well nourished according to the SGA assessment (score 6 or 7). Bioimpedance spectroscopy analysis demonstrated changes in body composition in both groups over the course of the study. The control group exhibited reduced lean tissue by the end of the study (50.2 ± 12.1 vs 51.8 ± 13.7%, *p* = 0.041) as well as increased overhydration (2.4 ± 2.0 v 1.7 ± 1.7 L, *p* = 0.001, Table 3). However, the intervention group observed a probable increase in dry weight (*p* = 0.05). Compared to the control group, dry weight possibly increased in the intervention group (+ 0.9 ± 1.6 vs − 0.2 ± 1.7 kg, *p* = 0.08) mostly attributable to a possible increase in lean tissue (+ 0.5 ± 3.3 vs − 1.6 ± 2.9%, *p* = 0.07) without change in hydration status (+ 0.2 ± 1.4 vs + 0.7 ± 0.8 L, *p* = 0.20). Similarly, handgrip strength decreased in the control group (29.7 ± 10.1 vs 31.2 ± 10.9 kg, *p* = 0.034) without change in the intervention group, but there was no clear difference between the deterioration seen in intervention and control groups (− 0.4 ± 3.4 vs − 1.5 ± 3.0 kg, *p* = 0.32). Analysis of 3-day food diaries did not show an increase in energy or protein intake (Table 3) suggesting a prevention of catabolism of muscle in the intervention group as the possible mechanism for changes in body composition as a result of improved bicarbonate levels. The normalised protein catabolic rate, however, remained stable (Table 3).

### Cardiovascular data

Haemodialysis sessions induced a significant increase in heart rate, and significant changes in ECG morphology with increased heart rate, longer PR, broader QRS and shorter QTc observed post-dialysis compared to pre-dialysis in the control group (*p* < 0.001 for each). In the intervention group, bicarbonate treatment did not have a significant impact on any of these changes, nor was there a significant effect on QT interval dispersion (Table 4). Although there was no between group difference in blood pressure during treatment weeks 13-16, blood pressures in the intervention group were higher during treatment than without, by 5.8/3.8 mmHg (systolic/diastolic, *p* = 0.005/0.021, Table 5). This was not explained by a corresponding change in inter-dialytic weight gain, which was similar (2.61 ± 0.97 vs 2.52 ± 1.13 kg, *p* = 0.46). One participant required an increase in the dose of one of his antihypertensive medications.

**Table 4:** Electrocardiogram changes in intervention and control groups

		No treatment		Week 5-8			Week 13-16			Final dose		
		Mean	SD	Mean	SD	p value^a^	Mean	SD	p value^a^	Mean	SD	p value^b^
**Intervention (N = 16)**												
Heart rate (s-1)	Pre HD	70.2	15.5	71.8	12.4	*0.18*	73.8	14.1	*0.83*	73.0	13.8	*0.98*
	Post HD	76.5	12.3	75.5	13.1	*0.22*	76.6	14.7	*0.59*	76.2	13.7	*0.24*
	HD effect	+ 6.3	8.8	+ 3.6	9.8	*0.83*	+ 2.8	9.7	*0.63*	+ 3.2	9.2	*0.15*
PR interval	Pre HD	173	34	177	42	*0.97*	173	35	*0.60*	175	36	*0.99*
	Post HD	163	29	169	34	*0.89*	162	25	*0.44*	165	28	*0.33*
	HD effect	−9.9	19.7	−7.8	14.7	*0.93*	−11.5	21.0	*0.74*	−9.6	16.8	*0.94*
QRS duration	Pre HD	94	17	92	14	*0.95*	94	16	*0.93*	94	16	*0.69*
	Post HD	97	15	94	15	*0.86*	96	16	*0.73*	96	16	*0.89*
	HD effect	+ 3.3	8.9	+ 2.3	5.3	*0.71*	+ 2.8	8.4	*0.58*	+ 2.6	8.0	*0.62*
Corrected QT	Pre HD	428	31	429	22	*0.08*	429	19	*0.21*	429	19	*0.46*
	Post HD	437	30	438	38	*0.38*	441	31	*0.29*	439	31	*0.51*
	HD effect	+ 8.8	22.4	+ 9.1	27.1	*0.51*	+ 11.7	28.8	*0.71*	+ 10.4	27.9	*0.79*
QT dispersion	Pre HD	35	15	34	12	*0.83*	41	13	*0.36*	40	12	*0.44*
	Post HD	38	13	33	12	*0.96*	34	12	*0.48*	34	12	*0.12*
	HD effect	+ 3.2	15.9	−1.7	12.6	*0.88*	−7.3	13.6	*0.18*	−5.7	13.9	*0.18*
**Control (N = 20)**												
Heart rate (s-1)	Pre HD	73.0	16.2	74.7	13.6		71.4	12.9				
	Post HD	81.7	17.1	78.9	14.8		76.6	14.8				
	HD effect	+ 8.7	12.0	+ 4.2	10.5		+ 5.2	11.1				
PR interval	Pre HD	179	39	174	44		179	40				
	Post HD	166	42	169	47		169	38				
	HD effect	−13.6	16.4	−5.7	13.9		−9.9	11.5				
QRS duration	Pre HD	92	18	93	18		95	21				
	Post HD	95	19	95	21		98	18				
	HD effect	+ 3.7	4.6	+ 2.4	6.4		+ 3.7	7.3				
Corrected QT	Pre HD	437	52	437	22		431	16				
	Post HD	446	34	443	30		446	28				
	HD effect	+ 8.9	44.6	+ 5.7	25.6		+ 14.9	20.8				
QT dispersion	Pre HD	36	12	35	12		36	12				
	Post HD	37	15	32	13		36	14				
	HD effect	+ 0.2	15.6	−3.9	13.0		−0.4	16.2				

Except where stated, measurements are in ms

HD haemodialysis

^a^Between groups (intervention vs control)

^b^Within intervention group (final dose vs no treatment)

**Table 5:** Blood pressure and inter-dialytic weight gain in intervention and control groups

	No treatment		Week 5-8			Week 13-16			Final dose		
	Mean	SD	Mean	SD	p value^a^	Mean	SD	p value^a^	Mean	SD	p value^b^
**Intervention (N = 16)**											
Pre HD systolic pressure	136	14	138	15	*0.53*	141	15	*0.95*	142	16	0.005*
Pre HD diastolic pressure	78	11	80	12	*0.44*	84	9	*0.65*	82	11	0.021*
ID weight gain (kg)	2.52	1.13	2.48	0.97	*0.15*	2.74	0.96	0.044*	2.61	0.97	*0.46*
**Control (N = 20)**											
Systolic pressure	143	18	141	16		141	18				
Diastolic pressure	84	12	83	11		82	11				
ID weight gain (kg)	2.07	1.28	1.93	1.17		1.96	1.13				

Except where stated, measurements are in mmHg

HD haemodialysis

ID inter-dialytic interval

^a^Between groups (intervention vs control)

^b^Within intervention group (final dose vs no treatment)

*Significant at p < 0.05

### Adherence and adverse events

Adherence to treatment varied between 67 and 100% with an average of 90.3% as measured using self-recorded medication diaries and remaining medication checks. Adverse events were no more common in the intervention group, and apart from the hypertension mentioned above, none were related to study treatment. No serious adverse events were observed in the intervention group during the study.

## Discussion

In this study, oral treatment with sodium bicarbonate substantially increased pre-dialysis bicarbonate levels, evening out the saw-toothed profile commonly present due to rapidly supplemented bicarbonate during dialysis. This is in turn, led to a significant reduction in inter-dialytic potassium gain, minimising episodes of clinically significant hyperkalaemia. The average effect size, a reduction in pre-dialysis potassium of 0.21 mmol/l, was in keeping with observational studies demonstrating the association between predialysis potassium and bicarbonate levels [8], and dose-responsive so that pre-dialysis potassium could be expected to reduce by approximately 0.1 mmol/l for every 1.0 mmol/l increase in bicarbonate.

The danger of high potassium pre-dialysis is frequently encountered in clinical practice, and well supported by observational data: in a study of over 70,000 patients, after adjustment for case mix and malnutrition parameters, a progressive increase in mortality risk was seen with higher pre-dialysis potassium starting at 5.6 mmol/l [4]. Low potassium post-dialysis is also harmful - though rarely measured. Risk can be inferred from studies of dialysate potassium, which reveal an increased risk of sudden cardiac death with low dialysate potassiums, for example in a study of 36,235 patients in 12 countries, an increased risk was observed with any dialysate potassium below 3.0 mmol/l (HR 1.17, 95% CI 1.01–1.37) [18]. The harm could be due to the impact low potassium dialysates have on post dialysis potassium levels or due to the abrupt change in potassium levels incurred over a dialysis session. Using low dialysate potassium therefore as a treatment for pre-dialysis hyperkalaemia may be counterproductive, and although in our study sodium bicarbonate was biochemically only half as effective as dialysate adjustment in reducing pre-dialysis potassium, as it doesn’t lower post-dialysis potassium, it may therefore be a safer and clinically superior approach.

Two other established approaches to reduce pre-dialysis hyperkalaemia which don’t exacerbate post-dialysis hypokalaemia, are increased dialysis frequency and dietary restriction. The former is effective though burdensome in terms of patient time and dependent on local provision, whereas the latter is in routine use for many patients but with limited effectiveness, partly due to poor adherence and difficulties achieving dietary restriction without reducing other aspects of diet quality. Medications which control inter-dialytic potassium gain would therefore be advantageous, and some have recently become available: patiromer, for example, binds potassium in the gastrointestinal tract, leading to an average reduction in pre-dialysis potassium of 0.50 mmol/l. This study therefore demonstrates that sodium bicarbonate is an inexpensive established treatment which also reduces potassium gain - although the effect size is modest, like patiromer, it appears to have a greater effect in those with clinically relevant hyperkalaemia. Therefore the clinical benefit may be larger than would be predicted by the average potassium reduction.

Another positive effect of oral sodium bicarbonate supplementation was in minimising muscle breakdown. Acidosis is a well described cause of decreased muscle synthesis, with low pre-dialysis bicarbonate levels strongly associated with indicators of catabolism, such as protein catabolic rate [19]. Studies investigating the nutritional impact of acidosis treatment in people on haemodialysis typically focus on correction through increasing dialysate bicarbonate content as opposed to supplementing oral bicarbonate. These studies have tended to be small, underpowered and lacking in a control group. Movilli et al. undertook a longitudinal (uncontrolled) study of oral bicarbonate supplementation (ranging 1-4 g) in 29 people on haemodialysis over 4 months which lead to reduced catabolism overall (calculated by normalised protein catabolic rate) and improved albumin synthesis in the less inflamed subgroup (hsCRP < 10 mg/L) [20]. The evidence in people on peritoneal dialysis is far stronger with a randomised, placebo controlled double blind study showing a significant improvement in its primary outcome of nutritional status and mid arm muscle circumference (surrogate measure for muscle mass) over 12 months of oral bicarbonate supplementation (0.9 g thrice daily) [21]. Our study observed important intervention group improvements in dry weight and lean tissue mass over the 12 weeks (by approximately 1%), suggesting that continuous bicarbonate replacement is effective in reducing catabolism, in view that observed energy and protein intakes were similar in both control and intervention groups. A comparable improvement in muscle mass (1 kg) was seen in a randomised placebo controlled study after 3 months in people on haemodialysis, in which the intervention group received 100 mg/week of nandrolone decanoate (human growth hormone) by intramuscular injection [22]. Our results also echo those observed in two randomised studies in people with Chronic Kidney Disease (CKD) stages 3-4 undergoing sodium bicarbonate oral supplementation: sodium bicarbonate reduced catabolism resulting in an increase in muscle mass (equivalent to 4%) over a 2 year time period [23]; and increase in total body mass by approximately 2.7% over 4 months in the increased sodium bicarbonate group [24]. As haemodialysis is a catabolic process, it is perhaps unsurprising that the degree of muscle and body mass gain in CKD is not mirrored in people on haemodialysis, and therefore of significant relevance that they are present at all.

Another possible acidosis mediated effect is impairing muscle strength, although the mechanism is unclear. Yenchek et al. observed a relationship in a cohort of 1544 well-functioning older people from the Health, Aging and Body Composition prospective study (with and without kidney disease) between lower bicarbonate levels 3 years post baseline and incident persistent functional limitation [25]. The findings in our study in relation to muscle strength are less clear. The control group had a higher baseline handgrip strength with a significant decline over the course of the study, not mirrored in the intervention group. However, a recent systematic review found no significant differences in handgrip strength in intervention groups receiving bicarbonate in people with CKD [26], nor in a large parallel-group, double blind, placebo controlled RCT in older people with stage 4 or 5 CKD not on dialysis although more studies are currently underway [27]. Therefore, the evidence so far points to correction of acidosis on dialysis leads to muscle mass preservation in people on HD, with further research required into acidosis correction and muscle strength and function.

Reliable changes in ECG morphology were observed immediately after haemodialysis sessions, as expected, though these changes were not altered by bicarbonate. Resting ECG however has limited sensitivity in general in detecting arrhythmia risk, and the ECG parameter thought to be the most predictive, QT dispersion, was not reliably altered by dialysis in this study, so this finding does not exclude the possibility of a clinically useful effect.

Adverse effects were minimal. Blood pressure was, as anticipated, increased by administration of sodium bicarbonate, and this adverse effect may offset some of the clinical benefit of treatment. These adverse effects are echoed in a recent systematic review in people with stages 3-5 CKD not on dialysis [26]. However, the modest effect size of increased blood pressure would allow compensation by a small increase in anti-hypertensive treatment, so that the clinical impact could be minimal.

This study has a number of limitations, in particular the small sample size, exacerbated by the withdrawal of some patients allocated to intervention before they started treatment. Recruitment failed to hit target despite screening and approaching all eligible patients within three satellite dialysis units. This was not a placebo controlled trial which may have impacted on some of the outcomes such as reporting and analysis of nutritional intake data. In addition, even with dose titration, many patients failed to achieve the predialysis bicarbonate target, mainly due to tablet burden which was cited as the key reason for reluctance to increase the dose. The study in people on peritoneal dialysis [21] used a formulation with a higher sodium bicarbonate content (0.9 g v 0.5 g) which would have made a significant difference to tablet burden and is important to implement in future studies. However, adherence was similar in the peritoneal dialysis and our study, despite the dose difference.

These factors somewhat limit the conclusions drawn, but the data nevertheless supports further exploration of an established but overlooked treatment, and provides information helpful in the design of future research, in particular alleviating some of the concern over blood pressure and fluid gain. The extent to which patients were reluctant to titrate treatment dose sufficiently might also be addressed in future studies with an alternative formulation for sodium bicarbonate.

## Conclusions

Oral sodium bicarbonate treatment was found to reduce potassium gain in the inter-dialytic period, in a dose-dependent manner, leading to reduced pre-dialysis potassium, without altering post-dialysis levels. Body composition changes following 12 weeks of treatment suggest that treatment may also preserve lean tissue mass in addition to potentially helping preserve muscle function. Oral sodium bicarbonate may reduce clinical consequences of hyperkalaemia in haemodialysis patients whilst also preventing nutritional decline, and further study of this simple treatment seems justified.

## Data Availability

The datasets generated and/or analysed during the current study are not publicly available due patient confidentiality but are available from the corresponding author on reasonable request.

## Abbreviations

HD: Haemodialysis
ECG: Electrocardiogram
SGA: Subjective Global Assessment
CKD: Chronic Kidney Disease
